# Circulating miRNA-181b-5p, miRNA-223-3p, miRNA-210-3p, let 7i-5p, miRNA-21-5p and miRNA-29a-3p in patients with localized scleroderma as potential biomarkers

**DOI:** 10.1038/s41598-020-76995-2

**Published:** 2020-11-19

**Authors:** Katarzyna Wolska-Gawron, Joanna Bartosińska, Marta Rusek, Małgorzata Kowal, Dorota Raczkiewicz, Dorota Krasowska

**Affiliations:** 1 Department of Dermatology, Venerology and Paediatric Dermatology, The Medical University of Lublin, 20-081 Lublin 11 Staszica St, Lublin, Poland; 2 Department of Cosmetology and Aesthetic Medicine, The Medical University of Lublin, Lublin, Poland; 3 Department of Pathophysiology, The Medical University of Lublin, Lublin, Poland; 4 SGH Warsaw School of Economics, Collegium of Economic Analysis, Institute of Statistics and Demography, Warsaw, Poland

**Keywords:** Epigenomics, Biomarkers, Clinical genetics, Connective tissue diseases, Epigenetics, Biomarkers, Molecular medicine, Pathogenesis

## Abstract

Localized scleroderma (LoSc) is a rare disease manifested by an inflammation and sclerosis of the skin. The latest studies focused on glycoprotein Krebs von den Lungen-6, surfactant protein-D, chemokine ligand 18 and dipeptidylpeptidase 4 as potential biomarkers of skin fibrosis in systemic scleroderma. Our study aimed to identify 6 miRNAs with elevated or decreased levels in 38 LoSc patients (31 females, 7 males) compared to healthy volunteers (HVs) and to correlate the selected miRNAs’ serum levels with the severity and the clinical symptoms of LoSc and some laboratory parameters with the selected miRNAs’ serum levels. The serum levels of miRNAs, i.e. miRNA-181b-5p, miRNA-223-3p, miRNA-21-5p, let 7i-5p, miRNA-29a-3p and miRNA-210-3p were significantly increased in the LoSc patients compared to the HVs. The level of let-7i increase in the female LoSc patients correlated negatively with BSA (r =  − 0.355, p = 0.049) and mLoSSI (r =  − 0.432, p = 0.015). Moreover, the female patients with inactive LoSc had significantly higher level of let-7i (2.68-fold on average) in comparison to those with active disease (p = 0.045). The exact role of those molecules has not been revealed in LoSc and a long-term longitudinal research is pivotal to confirm their prognostic value.

## Introduction

Localized scleroderma (LoSc) is a rare, autoimmune disorder that is manifested by an inflammation and sclerosis of the skin. LoSc is most frequent in middle-aged women (20–50 years old). According to a German classification, LoSc may be divided into 5 subtypes—limited, generalized, linear, deep, and mixed^1^. The clinical course of LoSc is diversified, based on its activity/severity, as well as expanse and depth of lesions^2,3^. Although the majority of LoSc cases have mild, transient course, more severe ones affect structures lying beneath the skin, leading to irreversible sequelae^2,3^. Delayed diagnosis may cause timelapse in therapy and, in consequence, functional disabilities and disfiguration^1^. A pivotal point in evaluating patients with LoSc is the assessment of disease activity/severity and the extent of tissue damage with the use of Localized Scleroderma Cutaneous Assessment Tool (LoSCAT)^2,4^. LoSCAT seems to be a promising, easy-to-use worksheet with its repeatability and consistency of assessment^4^. Current research is focusing on the discovery of useful biomarkers reflecting ongoing fibrosis that might improve early diagnosis of LoSc.

MicroRNAs (miRNAs, miRs) are small, noncoding RNA molecules that serve as negative regulators of gene expression at the posttranscriptional level^5,6^. They constitute a crucial switch of cell proliferation, differentiation, apoptosis, and immune response^5,6^. In the last decade, miRNA has attracted a tremendous interest as an essential epigenetic regulator of pathological events in fibrotic diseases. MicroRNAs enhance or inhibit fibrosis by aiming extracellular matrix proteins (ECM), connective tissue growth factor (CTGF), transforming growth factor-beta (TGF-β) pathway, epithelial-to-mesenchymal transition (EMT) or proliferation of myofibroblasts^7,8^. MiRNAs are relatively stable, detectable in tissues and body fluids (serum, plasma), which makes them promising biomarkers to monitor the course of the disease and therapy response^9^.

Results of current studies provide little evidence on the role of miRNA in LoSc. To the best of our knowledge, the expression levels of 5 miRNA (miRNA-155 and miRNA-483 miRNA-7, let-7, miRNA-196a) have been evaluated thus far in LoSc patients^10–15^. The aim of our study was to delineate miRNAs that may serve as biomarkers of LoSc, as well as to correlate the serum levels of the selected miRNAs with the severity and clinical symptoms of LoSc and some laboratory parameters.

## Results and discussion

In the light of previous studies, miRNA-155 and miRNA-483 are profibrotic miRNAs with increased serum level in patients with LoSc, whereas miRNA-7, let-7, and miRNA-196a stand for antifibrotic ones with down-regulated expression^10–15^. The authors have not found any significant correlations between the serum level and expression levels of the aforementioned miRNAs and severity and clinical symptoms of LoSc and some laboratory parameters^10–14^.

**Figure 1:**
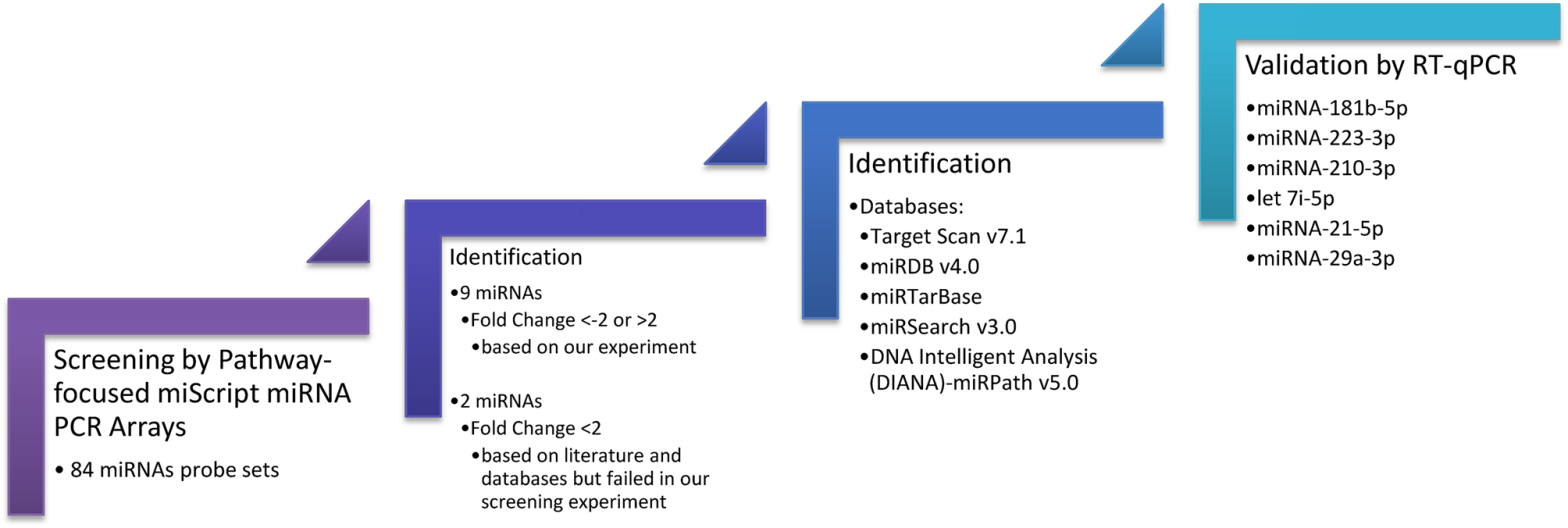
The strategy of miRNAs selection in our study.

For our analysis, we selected 4 miRNAs with ≥ 2.0-fold change—miRNA-181b-5p, miRNA-223-3p, let 7i-5p and miRNA-21-5p. Additionally, we chose miRNA-29a-3p and miRNA-210-3p with a fold change ≤ 2, failed in our screening experiment, due to their probable significance in diseases associated with fibrosis found in databases (Table 1). The strategy of miRNAs selection in our study is presented in Fig. 1.

**Table 1:** Summary of miRNA PCR array.

Position	Mature ID	Fold Change	Position	Mature ID	Fold Change	Position	Mature ID	Fold Change
A01	hsa-miR-142-5p	0.09	C09	hsa-miR-125b-5p	0.09	F05	hsa-let-7b-5p	1.21
A02	hsa-miR-9-5p	0.09	C10	hsa-miR-99a-5p	0.09	F06	hsa-miR-19b-3p	4.44
A03	hsa-miR-150-5p	0.09	C11	hsa-miR-28-5p	0.09	F07	hsa-miR-17-5p	0.84
A04	hsa-miR-27b-3p	0.09	C12	hsa-miR-320a	0.19	F08	hsa-miR-93-5p	0.74
A05	hsa-miR-101-3p	0.12	D01	hsa-miR-125a-5p	0.09	F09	hsa-miR-186-5p	0.09
A06	hsa-let-7d-5p	0.40	D02	hsa-miR-29b-3p	0.09	F10	hsa-miR-196b-5p	0.09
A07	hsa-miR-103a-3p	0.16	**D03**	**hsa-miR-29a-3p**	**0.66**	F11	hsa-miR-27a-3p	0.12
A08	hsa-miR-16-5p	2.17	D04	hsa-miR-141-3p	0.21	F12	hsa-miR-22-3p	0.09
A09	hsa-miR-26a-5p	1.01	D05	hsa-miR-19a-3p	0.09	G01	hsa-miR-130a-3p	0.09
A10	hsa-miR-32-5p	0.09	D06	hsa-miR-18a-5p	0.09	G02	hsa-let-7c-5p	0.16
A11	hsa-miR-26b-5p	0.44	D07	hsa-miR-374a-5p	0.09	G03	hsa-miR-29c-3p	0.15
A12	hsa-let-7 g-5p	0.04	D08	hsa-miR-423-5p	0.10	G04	hsa-miR-140-3p	0.09
B01	hsa-miR-30c-5p	0.09	D09	hsa-let-7a-5p	1.13	G05	hsa-miR-128-3p	0.09
B02	hsa-miR-96-5p	0.09	D10	hsa-miR-124-3p	12.82	G06	hsa-let-7f.-5p	0.35
B03	hsa-miR-185-5p	0.09	D11	hsa-miR-92a-3p	3.23	G07	hsa-miR-122-5p	0.40
B04	hsa-miR-142-3p	0.11	D12	hsa-miR-23a-3p	1.23	G08	hsa-miR-20a-5p	0.56
B05	hsa-miR-24-3p	1.40	E01	hsa-miR-25-3p	1.68	G09	hsa-miR-106b-5p	0.12
B06	hsa-miR-155-5p	0.09	E02	hsa-let-7e-5p	0.13	G10	hsa-miR-7-5p	0.09
B07	hsa-miR-146a-5p	0.09	E03	hsa-miR-376c-3p	0.09	G11	hsa-miR-100-5p	0.09
B08	hsa-miR-425-5p	0.09	E04	hsa-miR-126-3p	0.70	G12	hsa-miR-302c-3p	0.09
**B09**	**hsa-miR-181b-5p**	**25.99**	E05	hsa-miR-144-3p	0.12	H01	cel-miR-39-3p	0.09
B10	hsa-miR-302b-3p	0.09	E06	hsa-miR-424-5p	0.09	H02	cel-miR-39-3p	0.09
B11	hsa-miR-30b-5p	0.09	E07	hsa-miR-30a-5p	0.14	H03	SNORD61	0.09
**B12**	**hsa-miR-21-5p**	**2.01**	E08	hsa-miR-23b-3p	0.16	H04	SNORD68	0.09
C01	hsa-miR-30e-5p	0.05	E09	hsa-miR-151a-5p	0.09	H05	SNORD72	0.09
C02	hsa-miR-200c-3p	0.09	E10	hsa-miR-195-5p	1.51	H06	SNORD95	0.09
C03	hsa-miR-15b-5p	0.99	E11	hsa-miR-143-3p	0.09	H07	SNORD96A	0.09
**C04**	**hsa-miR-223-3p**	**6.23**	E12	hsa-miR-30d-5p	0.10	H08	RNU6-6P	0.09
C05	hsa-miR-194-5p	0.09	F01	hsa-miR-191-5p	0.60	H09	miRTC	1.22
**C06**	**hsa-miR-210-3p**	**0.04**	**F02**	**hsa-let-7i-5p**	**14.52**	H10	miRTC	1.35
C07	hsa-miR-15a-5p	0.09	F03	hsa-miR-302a-3p	5.90	H11	PPC	0.05
C08	hsa-miR-181a-5p	0.09	F04	hsa-miR-222-3p	0.19	H12	PPC	0.05

MiRNAs selected for further studies by RT-qPCR are presented in bold.

*Position* position on the plate for specific miRNA, *Mature ID* mature miRNA ID, *Fold Change* 2^−ΔΔCt^, cel-*miR-39-3p and cel-miR-39-3p* miScript primer used for alternative data normalization using exogenously spiked Syn-cel-miR-39 miScript miRNA mimic, *SNORD61, SNORD68, SNORD72, SNORD95, SNORD96A, RNU6-6P 6* miScript PCR controls used data normalization using the ΔΔCT method of relative quantification, *miRTC* reverse transcription control used for assessment of reverse transcription performance, *PPC* positive PCR control used for assessment of PCR performance.

**Figure 2:**
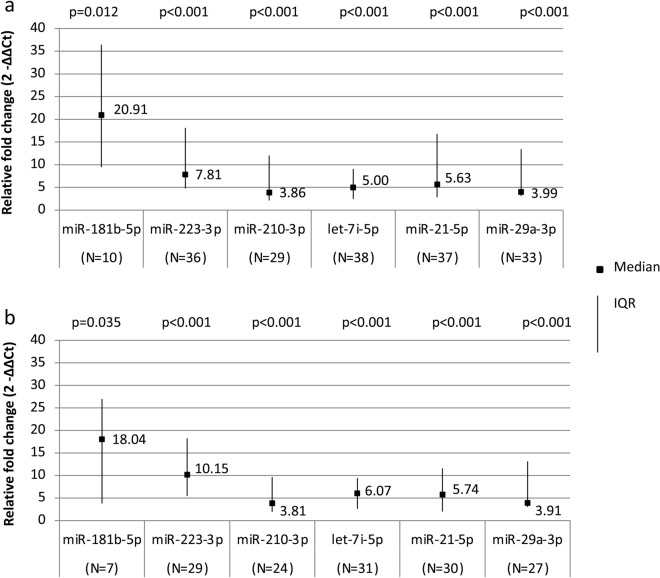
miRNAs’ relative fold change (2^−ΔΔCt^) in all localized scleroderma patients compared to healthy volunteers (**a**) and in female localized scleroderma patients compared to healthy volunteers (**b**). p for Mann–Whitney’s test, *LoSc* localized scleroderma, *IQR* interquartile range.

Firstly, we found that the serum levels of the six studied miRNAs, i.e. miRNA-181b-5p, miRNA-223-3p, miRNA-21-5p, let 7i-5p, miRNA-29a-3p and miRNA-210-3p were significantly increased in all the LoSc patients (both female and male) (Fig. 2a) and the female LoSc patients (Fig. 2b) compared to HVs.

The serum level of miRNA-181b-5p in all the studied LoSc patients compared to the HVs was increased 20.91-fold on average (p = 0.012). The up-regulation of miRNA-181b has already been reported in patients with hypertrophic scars^16,17^. Moreover, according to Wuttge et al., patients with diffuse cutaneous SSc (dcSSc) presented higher plasma levels of miRNA-181b compared to those with limited cutaneous SSc (lcSSc)^18^. Thus, it is plausible that miRNA-181b might be associated with severe types of LoSc manifested by generalized skin/deeper tissue involvement, complicated by extracutaneous manifestations.

Our study revealed that the serum level of miRNA-223-3p was increased 7.81-fold in the LoSc patients compared to the HVs (p < 0.001). The up-regulation of miRNA-223 in other fibrotic disorders (cardiac fibrosis, liver fibrosis, peritoneal fibrosis, SSc) and the elevation of miRNA-223 plasma level in autoimmune diseases (e.g. lupus erythematosus, rheumatoid arthritis) have already been delineated^19–23^. Liu et al. reported that miRNA-223 mimics enhanced cell proliferation and production of collagen I, collagen III, α-SMA (alpha-smooth muscle actin) in cultured cardiac fibroblasts^20^. α-SMA is overproduced by fibroblasts and pointed out to be an indicator of myofibroblast differentiation^15^. Both persistent fibroblast proliferation and differentiation into myofibroblasts result in ECM synthesis and deposition—a crucial point in the pathogenesis of LoSc^15^.

Our study showed that the serum level of miRNA-21-5p in the studied LoSc patients compared to the HVs was elevated 5.63-fold on average (p < 0.001). Our results are consistent with other studies results displaying the up-regulation of miRNA-21 in SSc, idiopathic pulmonary fibrosis (IPF) and keloids^24–26^. MiRNA-21 plays a key role in fibrosis through inhibition of apoptosis, regulation of ECM production, and EMT^27^. According to Jafarinejad et al., the up-regulation of miRNA-21 enhance Bcl-2 (anti-apoptotic protein) expression in fibroblasts and decrease the Bax: Blc-2 expression ratio significantly, leading to apoptosis resistance^24,26^. Moreover, the up-regulation of miRNA-21 attenuates the expression of Smad7 (SMAD Family Member 7)—a downstream inhibitor of TGF-β/Smad signaling pathway^26,27^.

We found that the serum level of let-7i-5p was increased 5.0-fold in the LoSc patients compared to the HVs (p < 0.001). Some authors have already shed light on the relevance of let-7i in fibrotic diseases. Wang et al. reported that let-7i acts as a novel regulator of cardiac fibrosis and inflammation by suppressing the expression of interleukin-6 and multiple collagens^28^. Furthermore, Makino et al. demonstrated that let-7i expression in 7 LoSc skin biopsies and 7 SSc skin samples were significantly decreased, compared with 7 healthy skin biopsies and 5 keloid skin samples^11^. This demonstrates that the level of let-7i may vary between different biological materials (skin tissue/ body fluids).

Furthermore, we demonstrated that the serum level of miRNA-29a-3p was increased 3.99-fold in the LoSc patients compared to the HVs (p < 0.001). These results are in concordance with the studies published by Szemraj-Rogucka et al. and Roncarati et al.^29,30^. These studies showed the elevated plasma level of miRNA-29a in hypertrophic cardiomyopathy patients that correlated with fibrosis^29^. Similar results were presented by Kawashita et al. who observed the increased level of miRNA-29a in patients with SSc^31^. Since other studies, performed on skin biopsies, showed the down-regulation of miRNA-29a in the diseases manifesting with fibrosis (i.e. keloids, hypertrophic scars, pulmonary fibrosis, SSc), it might suggest that the level of miRNA-29a may vary between different biological materials (skin tissue/body fluids)^32–34^.

In addition, our study revealed that the serum level of miRNA-210-3p in the LoSc patients compared to the HVs was increased 3.86-fold (p < 0.001). It is in concordance with the results presented by Oak et al. and Bodempudi et al.^35,36^. These authors found the expression of miR-210 to be increased in patients with rapidly progressive IPF who have typically experienced hypoxia^35,36^. Furthermore, the increased level of miRNA-210 has been exhibited in other autoimmune diseases (i.e. rheumatoid arthritis, lupus erythematosus)^37^.

Moreover, we attempted to correlate the serum levels of the aforementioned miRNAs with the severity and the clinical symptoms of LoSc and some laboratory parameters in all the study group (both the female and male LoSc patients) and in only the female LoSc patients. In all the study group we found that the studied miRNAs’ serum levels (miRNA-181b-5p, miRNA-223-3p, miRNA-21-5p, let 7i-5p, miRNA-29a-3p and miRNA-210-3p) did not correlate with age, gender, duration of the disease, the clinical subtype of LoSc, BSA, mLoSSI, LoSDI, disease activity, the presence of sclerotic areas, erythematous patches without sclerosis, patches with a lilac ring, hyperpigmented patches, pruritus, extracutaneous manifestations, distribution of lesions, positive ANA or RF as well as abnormal ESR and CRP levels (p > 0.05).

**Figure 3:**
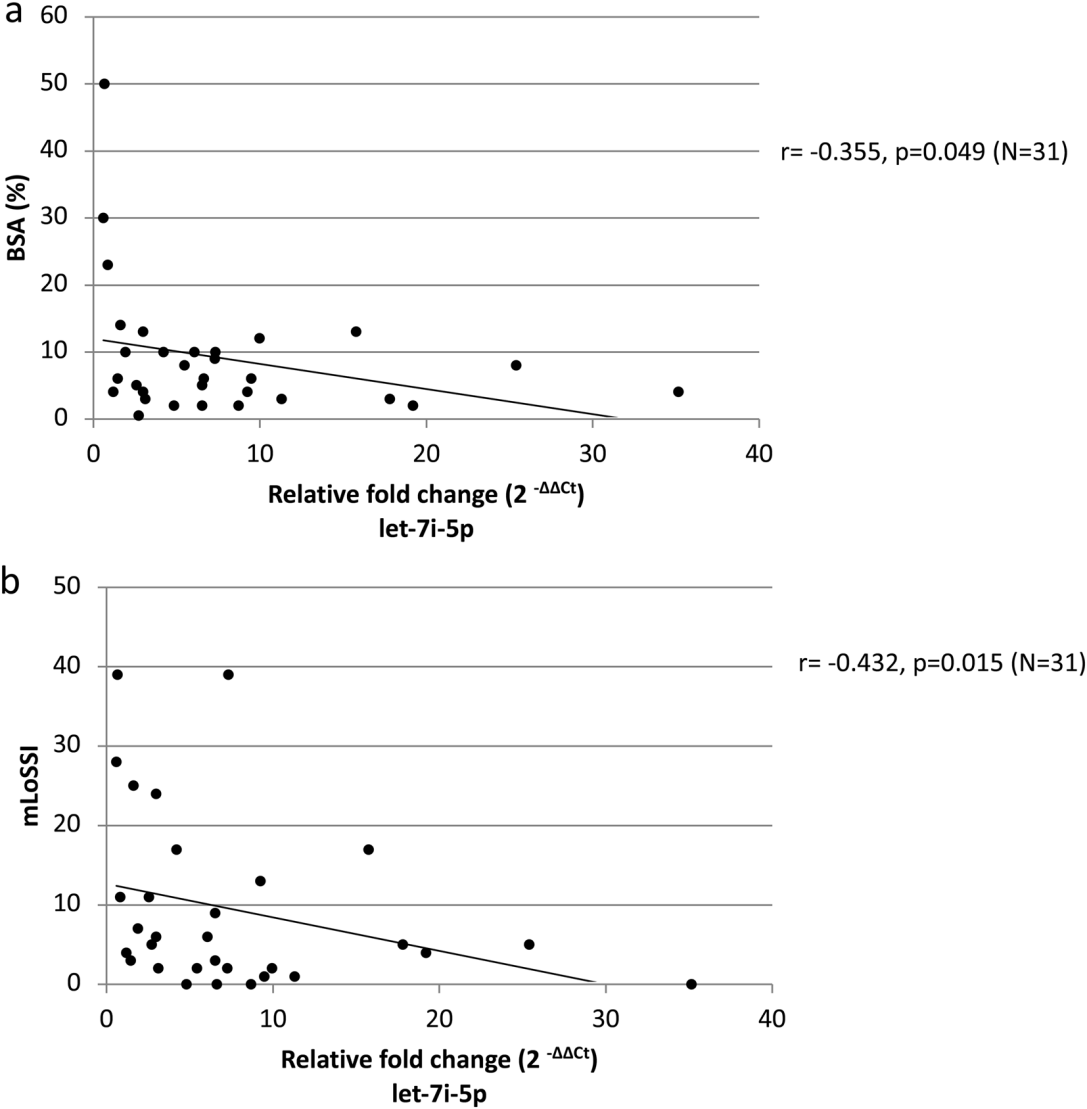
Relative fold change (2^−ΔΔCt^) of let-7i-5p versus BSA (**a**) and mLoSSI (**b**) in female localized scleroderma patients. *r* Spearman’s correlation coefficient, *BSA* body surface area, *mLoSSI* modified Localized Scleroderma Skin Severity Index.

**Figure 4:**
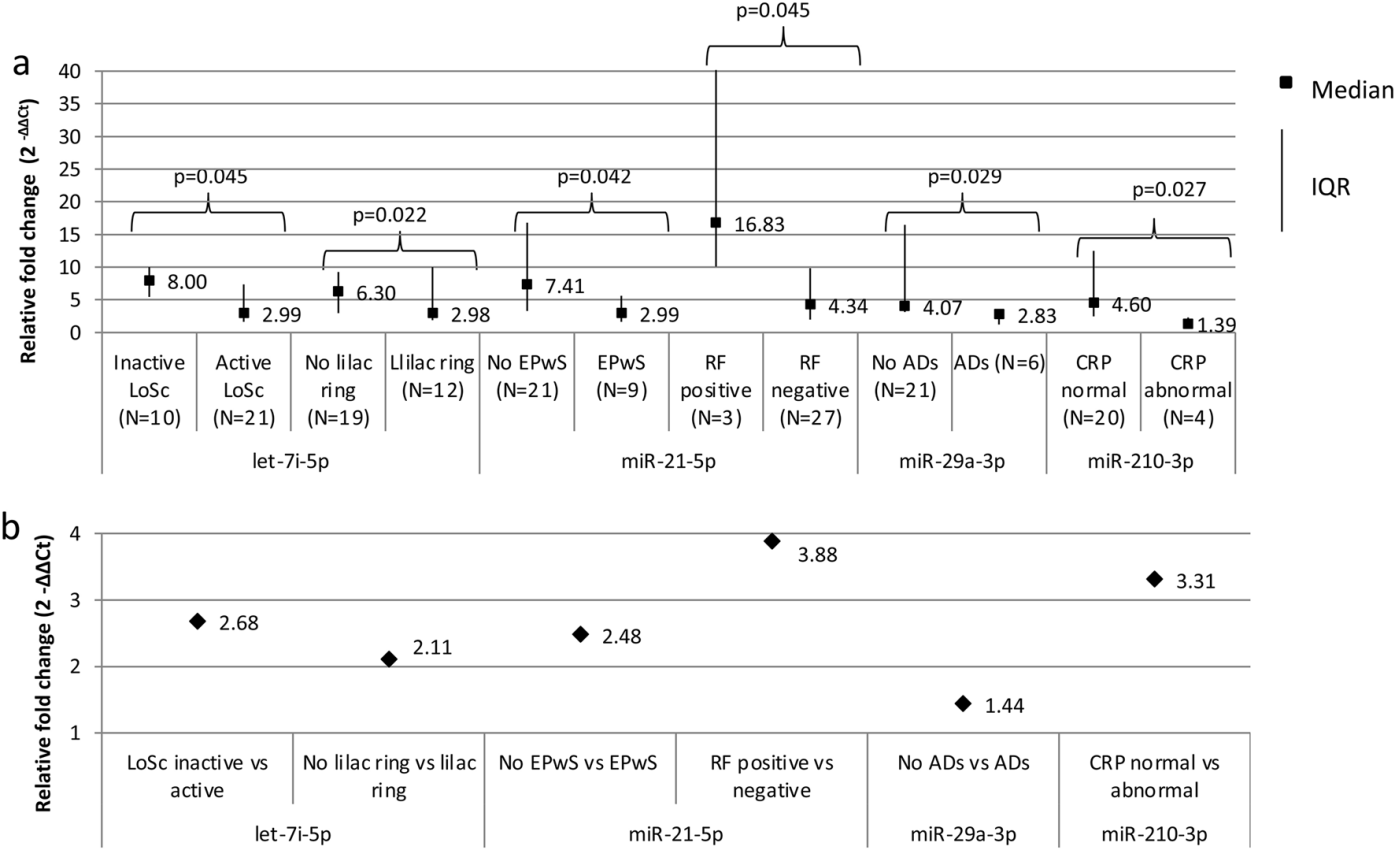
Statistically significant correlations between miRNAs’ relative fold change (2^−ΔΔCt^) and clinical characteristics of the female localized scleroderma patients. (**a**) Relative fold change in patients versus healthy volunteers. (**b**) Relative fold change in patients compared between two groups. p for Mann–Whitney’s test, *IQR* interquartile range, *LoSc* localized scleroderma, *CRP* C-reactive protein, *RF* rheumatoid factor, *EPwS* erythematous patches without sclerosis, *ADs* concurrent autoimmune diseases.

However, in the female LoSc patients we have found some significant correlations between some miRNAs’ serum levels and the severity, the clinical symptoms of LoSc and some laboratory parameters of the LoSc characteristics (Figs. 3, 4). The level of let-7i increase in the female LoSc patients correlated negatively with BSA (r =  − 0.355, p = 0.049; Fig. 3a) and mLoSSI (r =  − 0.432, p = 0.015; Fig. 3b), i.e. the higher the severity of the disease measured by BSA and mLoSSI, the lower the increase level of let-7i. Moreover, the female patients with inactive LoSc had significantly higher level of let-7i (2.68-fold on average) in comparison to those with active disease (p = 0.045). In the female LoSc patients who had patches without lilac ring, significantly higher level of let-7i (2.11-fold on average) than in the female patients with lilac ring (a feature of disease activity) was observed (p = 0.022) (Fig. 4). These results suggest that the higher increase level of let-7i co-exist with less severe LoSc. Makino et al. in their group of SSc patients observed that the serum let-7 level inversely correlated with the severity of skin sclerosis^11^.

**Figure 5:**
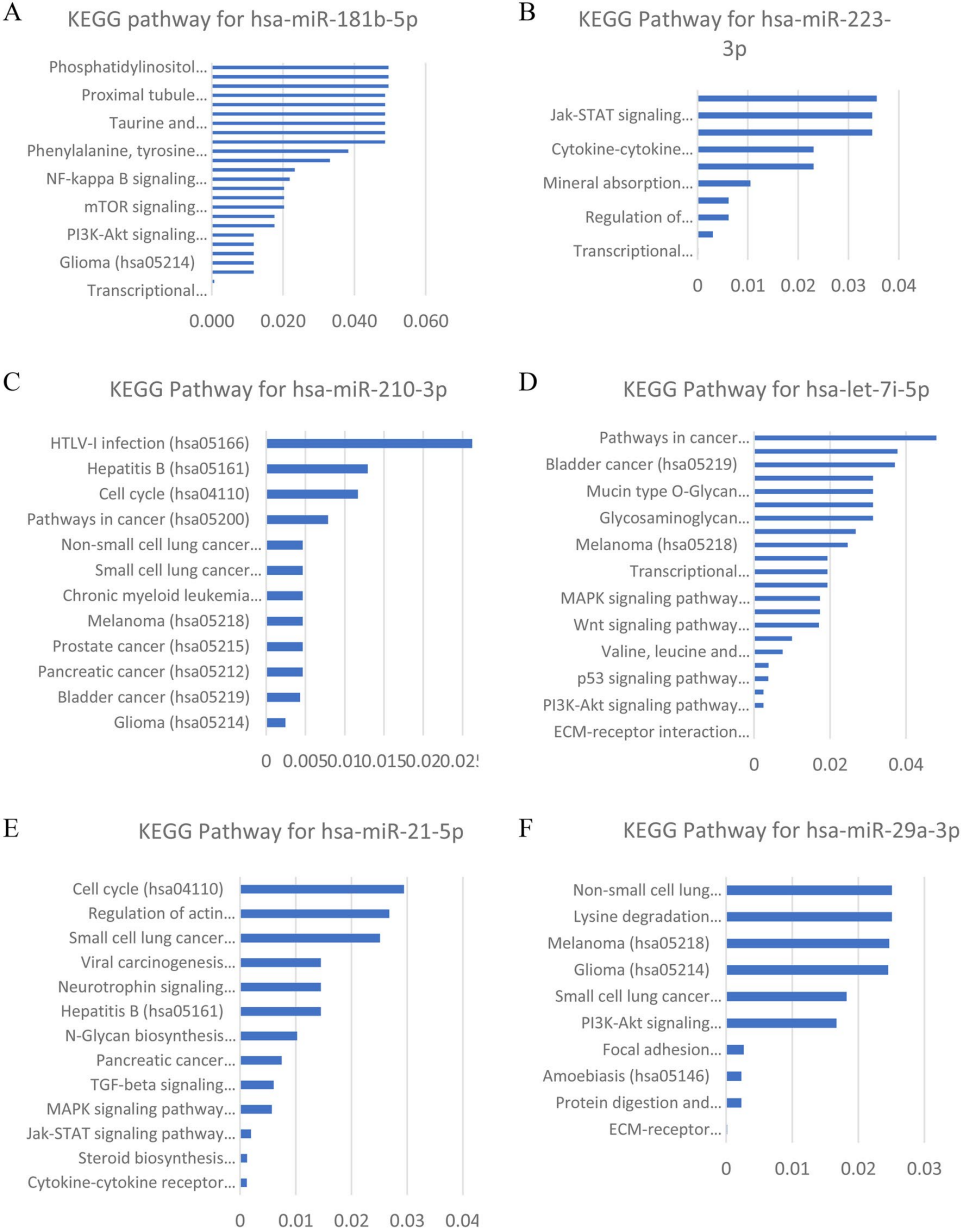
Enrichment analysis. (**A**) The KEGG pathway analysis of hsa-181b-5p; (**B**) the KEGG pathway analysis of hsa-miR-223-3p; (**C**) the KEGG pathway analysis of hsa-210-3p; (**D**) the KEGG pathway analysis of hsa-let-7i-5p; (**E**) the KEGG pathway analysis of hasmiR-21-5p; (**F**) the KEGG pathway analysis of hsa-29a-3p. The enrichment score is expressed as –log (p value)^39–42^.

Furthermore, our study revealed that higher level of miRNA-21 was observed in the female LoSc patients without erythematous patches without sclerosis (2.48-fold on average) than in those with erythematous patches without sclerosis (a marker of the inflammatory stage of the disease) (p = 0.042), indicating that miRNA-21 serum level is inversely correlated with inflammation (Fig. 4). We also demonstrated that the level of miRNA-21 was higher in the female LoSc patients with positive RF (3.88-fold) than in those with negative RF (p = 0.045) (Fig. 4). Additionally, our study showed a higher level of miRNA-29a in the female LoSc patients without the presence of concomitant autoimmune diseases (1.44-fold on average) than in those with autoimmune diseases (p = 0.029), as well as a higher level of miRNA-210 in female LoSc patients with the normal CRP level (3.31-fold on average) than in those with abnormal CRP level (a marker of the inflammatory stage of the disease) (p = 0.027) (Fig. 4). Anti-inflammatory properties of miRNA-210 have been already reported by Qi et al.^38^. Computational prediction of miR-181b-5p, miR-223-3p, miR-210-3p, let 7i-5p, miR-21-5p, and miR-29a-3p, using the aforementioned algorithms, generates a long list of potential targets and pathways involved in the process of fibrosis (Table 2, Fig. 5)^39–42^.

**Table 2:** Predicted target genes for hsa-miR-181b-5p, hsa-miR-223-3p, hsa-miR-210-3p, hsa-let 7i-5p, hsa-miR-21-5p, and hsa-miR-29a-3p.

Database miRNA	Target Scan v7.1 (https://www.targetscan.org)	miRDB v4.0 (https://mirdb.org/miRDB/)	DNA Intelligent Analysis (DIANA)-miRPath v5.0 software, based on the data from Ensembl v69 and miRBase v18 (https://diana.imis.athena-innovation.gr)
hsa-miR-181b-5p	OSBPL3, IL2, GSKI, KLF6, S1PR1	S1PR1, ADAM11, TGFBRAP1, LOX, ADAMTS18, TNF, ADAMTS6, TAB3, OSBPL2, ADAMTS5, ESM1, TAB2, TGFBR2, ADAMTSL1, PPARA, MMP14, ECT2L, ADAMTS19, ADAM28, TGFBI, COL6A3, SNAI2, WNT16, WIF1, CTNNA1, COL16A1, MMP7	PPP2R5E, CREB5, DDIT4, PIK3R3, F2R, SOS1, PTEN, SPP1, BCL2L11, RPS6KB1, CAMK2D, CRK
hsa-miR-223-3p	SEPT2, SEPT10, ECT2	SMAD1, TGFBR3, ECT2, MMP16, FGFR2, FGF2, ADAMTS15	PRLR, SPRED1, CBLB, LIF, PIK3R3, OS1, PIAS2, IL6ST, STAT1, PPP2R5E, CREB5, IGF1R, DDIT4, F2R, FOXO3, PKN2, FGFR2, RELA, PTEN, SPP1, BCL2L11, RPS6KB1
hsa-miR-210-3p	FGFRL1, KLF12	FGFRL1, WLS	
hsa-let 7i-5p	TGFBR1, ADAMTS8, APBB3	ADAMTS15, ADAMTS8, COL3A1, WNT9B, COL4A2, COL1A2, FGF11, COL4A1, HIF1AN, COL27A1, COL4A6, COL4A3BP, COL5A2	TSC1, MYB, NRAS, CCND2, COL27A1, IGF1R, COL3A1, RPS6KB2, TP53, GHR, COL1A1, INSR, NGF, COL1A2, ITGA7, COL4A6, CDKN1A, OSMR, COL5A2, COL4A1, IL6R, ACTB, MAPK8, FLNA, COL1A2, PORCN, FZD4, SENP2, NFAT5, GPC4, WNT9A, TGFBR1, NRAS, MAP4K3, MAP3K1, CSP3, RASGRP1, NGF, FAS, MEF2C, DUSP1
hsa-miR-21-5p	FGF18, TGFBI, TIMP3, SMAD7, FRS2, KLF6, ESM1, FGF7, AGO2, TGFBR2, KLF3, COQ10B, TGFB2	TGFBI, FGF18, SMAD7, ADAMTS3, TGFB2, COL4A1	ACVR2A, SMAD7, TGFBR2, BMPR2, COL4A1
hsa-miR-29a-3p	COL1A1, COL3A1, ELN, COL1A2, COL11A1, ADAMTS9, ADAMTS2, COL2A1, COL5A3, COL4A5, COL4A1, ADAMTS17, COL5A2, COL4A4, COL21A1, COL7A1, COL9A1, LOXL2, HAPLN3, COL19A1, ADAMTS10, COL15A1, VEGFA, COL27A1, COL5A1, ADAMTS7, WISP1, COL22A1, COL8A1, COL4A2, COL6A2, MMP16, ADAM19, ADAM12, EMP2, HAPLN1, COL4A3, EMP1, CTNND1, COL4A6, FRS2, COL25A1, COL16A1	COL5A3, COL5A1, COL3A1, COL11A1, ADAMTS9, COL19A1, ADAMTS6, COL4A1, COL1A1, COL7A1, ADAMTS17, HIF3A, COL5A2, COL2A1, ADAMTS2, COL9A1, LOX, VEGFA, FRAT2, COL4A4, ADAMTS7, COL15A1, COL4A2, COL22A1, COL1A2, LOXL2, COL25A1, ADAMTS10, COL4A6, COL4A5, COL27A1, COL4A3, ADAM12, COL6A1, COL6A2, ADAM22, COL6A6, CTNNBIP1, ADAM23, TGFB2	COL27A1, COL3A1, COL2A1, COL4A2 COL5A1, COL1A1, COL4A3, COL4A4 COL1A2, LAMC1, COL11A1, COL6A3 COL4A6, LAMA2, COL5A3, COL5A2 COL4A1, CAV2, AKT2, PDGFB, PIK3R1, LAMC1, IGF1, AKT3, PDGFC, PTEN, PDGFA

miRNA181-5p is predicted to target S1PR1 (sphigosine-1-phosphate receptor 1), KLF6 (Krueppel-like factor 6), BCL2L11 (Bcl-2-like protein 11), OSBPL3 (oxysterol binding protein-like 3), PTEN (phosphatase and tensin homolog), LOX (lysyl oxidase family of enzymes), IL-2 (interleukin 2), as well as NF-kappa B signaling pathway and PI3K-Akt signaling pathway. Sphingosine 1-phosphate (S1P) is a bioactive sphingolipid involved in the profibrotic inflammatory process through i.a. hematopoietic stem cells (HSCs) proliferation and differentiation to myofibroblasts^43^. S1P is synthesized by sphingosine kinases (SphKs) and acts through S1P specific cell surface receptors (S1PR1–5)^43^. Overexpression of S1PR1 has already been reported in cardiac hypertrophy and fibrosis through angiotensin II and interleukin-6^44^. Moreover, González-Fernández et al*.* delineated that targeting SphKs/S1P/S1P receptors signalling pathway has a very promising therapeutic potential in the treatment of hepatic fibrosis^43^. KLF6 is the transcription factor that plays roles in differentiation, development, apoptosis, and angiogenesis^45^. It activates a number of genes crucial for the development of liver fibrosis, including collagen 1, TGF-β1 and TGF- β1 receptors types I and II^45^. Starkel et al*.* reported that oxidative stress, KLF6 and TGF-β1 up-regulation discriminate non-alcoholic steatohepatitis advancing to fibrosis from uncomplicated steatosis in rats^46^. Furthermore, accumulating evidence demonstrates that KLF6 is involved in the occurrence of oral submucous fibrosis^47^. BCL2L11, also known as BIM, is a pro-apoptotic member of the B cell CLL/lymphoma 2 protein family and is an essential modulator of apoptosis^48^. In the course of fibrotic diseases, the excessive collagen deposition leads to increased ECM stiffness. Myofibroblasts activated by ECM stiffness are prepared for apoptosis by death signals such as the BCL2L11. Upregulation of BIM results in expression of anti-apoptotic proteins to provide myofibroblast survival^49^. OSBPL3 was delineated to increase in advanced stages of non-alcoholic fatty liver disease/non-alcoholic steatohepatitis leading to fibrosis^50^. PTEN is a lipid/protein phosphatase that negatively regulates proliferation by inhibiting the integrin–PI3K/Akt pathway^51^. Loss of PTEN expression has already been demonstrated in patients with dsSSc and IPF^51,52^. LOX is a family of ECM cross-linking enzymes that have been disclosed to play a crucial role in fibrogenesis^53^. Increased LOX gene expression has been described in IPF tissue compared with healthy controls in two independent studies^53,54^.

miRNA-223-3p is anticipated to target ETC2 (epithelial cell transformation-2), SMAD1, FGFR2 (fibroblast growth factor 2 receptor), FGF2 (fibroblast growth factor 2), FOXO3 (Forkhead box O3), SPP1 (osteopontin), MMP16 (matrix metalloproteinase 16), PTEN, BCL2L11, and JAK-STAT signalling pathway. ETC2, a factor with established roles in cytokinesis and extracellular-signal-regulated kinase (ERK) signalling, was reported to be increased in IPF^55^. SMAD1 is a protein mediating TGF-b signalling, activated by α-SMA, that was delineated to be elevated in the skin of patients with SSc i liver fibrosis^56^. FGF2 and FGFR2 are profibrogenic factors that may support the proliferation and activation of kidney fibroblasts, which contribute to the development of renal fibrosis^57^. FOXO3 is an important integrator of profibrotic signaling in lung fibrosis and was found to be downregulated in IPF myofibroblasts^58^. SPP1, a protein with proinflammatory and profibrotic properties, has already been demonstrated to take part in cardiac and lung fibrosis^59,60^. MMP16 protein activates MMP2 protein which in turn degrades type III collagen and was demonstrated to be decreased in the myocardial fibrosis^61^.

Databases predict that miRNA-210-3p targets FGFRL1 (fibroblast growth factor receptor-like 1), and WLS. FGFRL1, also known as FGFR5, is the atypical receptor that lacks an intracellular kinase domain and binds to FGF ligands (binds FGF3, FGF4, FGF8, FGF10, and FGF22). The FGF8/FGFRL1 complex regulates development of nephrons by controlling the mesenchymal-to-epithelial transition^62^. Modifications in the Wnt signaling pathway have been implicated in liver fibrosis^63^. Shutdown of Wnt secretion (WLS) from stellate cells is pivotal for development of liver fibrosis following hepatobiliary injury^63^.

Let-7i is foreseed to target collagens, NGF (nerve growth factor), HIF1AN (hypoxia-inducible factor 1-alpha inhibitor), TGFBR1 (transforming growth factor beta receptor 1), ADAMTS8 (ADAM metallopeptidase with thrombospondin type 1 motif 8), IL6R (interleukin 6 receptor), as well as PI3K signalling pathway, p53 signalling pathway, MAPK signalling pathway, and ECM-receptor interaction. NGF is a polypeptide which, in addition to its effect on nerve cells, is considered to play a function in inflammatory responses and tissue repair^64^. Fibroblasts express and release NGF protein, which may consequently mediate proliferation and hypertrophy. Overexpression of NGF has already been disclosed in SSc patients^65^. Chen et al*.* found miRNA-184 as a critical mediator to promote the renal fibrosis by targeting HIF1AN^66^. ADAMTS8 is a member of the ADAMTS family which is involved in a various of functions including migration, adhesion, proliferation, and growth factor signalling^67^. Increased ADAMTS8 protein levels were revealed in linear localized scleroderma lesions^67^.

Databases predict that miRNA-21-5p target collagens, ESM1 (endothelial cell-specific molecule 1), AGO2 (argonaute 2), TIMP3 (tissue inhibitor of metalloproteinases), BMPR2 (bone morphogenic protein receptor 2), and JAK-STAT signaling pathway. ESM1, also known as endocan, is reported to play a role in the pathogenesis of inflammation, endothelial dysfunction and vascular disorders^68^. Accumulating evidence suggest that elevated ESM1 expression promotes kidney fibrosis by inducing the endothelial to mesenchymal transition in a mouse model^68^. Moreover, Oak et al*.* revealed that AGO2, an essential component of the miRNA processing RISC (RNA-induced silencing complex) complex, was expressed at lower levels in rapidly progressive IPF biopsies compared with both normal and slowly progressive IPF biopsies^69^. According to Kassari et al*.* loss of tissue TIMP3, a key inhibitor of ECM remodeling, enhances renal fibrosis^70^. BMPR2 is a member of the TGF-β family that has a major role in suppressing TGF-β signaling^71^. Ning-Yuan et al*.* disclosed reduced BMPR2 expression in patients with IPF compared to healthy controls^71^.

miRNA-29a in anticipated to target collagens, CTNNBIP1 (catenin-beta interacting protein 1), LAMC1 (laminin gamma-1), AKT3 (Akt kinase isoform 3), CAV2 (caveolin 2), CTNND1 (catenin delta 1), WISP1 (WNT1-inducible-signaling pathway protein 1), PI3K signaling pathway, and ECM-receptor interaction. CTNNBIP1 plays an important role in the integration of cell adhesion, motility, cell death, and suppresses Wnt/β-catenin signaling^72^. LAMC1 is a core structural protein present in the basement membrane of several organs and released by MMP-9^73^. Nielsen et al*.* hypothesized that dysregulation of LAMC1 remodeling could be associated with a higher risk of renal fibrosis^73^. AKT3 is a serine/threonine-protein kinases which regulate many processes including metabolism, proliferation, cell survival, growth and angiogenesis^74^. Wang et al*.* reported that knockdown of AKT3 inhibited α-SMA and collagen I, and enchanced apoptosis preventing liver fibrosis^74^. Latest evidence suggest that CAV2 overexpression strongly reduce the expression of fibrotic markers, such as connective tissue growth factor, α-SMA, fibronectin, SMAD2/3^75^. Yang et al*.* postulated that suppressing CAV2 expression lead to hepatic fibrosis by promoting TGF-β pathway^75^. Furthermore, Deng et al. reported that CTNND1 overexpression increased the protein expression of collagen I, and α‐SMA. WISP1 is a matricellular protein encoded by a WNT target gene^76^. Increased expression of WISP1 in alveolar cells was revealed in patients with IPF^76^.

The comprehensive applicability of miRNAs may help early diagnosis of LoSc, preventing irreversible sequelae like e.g. contractures, limb asymmetry or scoliosis. Furthermore, miRNAs can have therapeutic significance and may possibly modulate skin fibrosis by enhancing the expression of anti-fibrotic miRNAs or decreasing the expression of profibrotic miRNAs.

Limitations of the study included a small cohort (N = 38) and insufficient diversity of LoSc clinical subtypes (lack of deep subtype).

In conclusion, our preliminary study showed significant increased level of miRNA-181b-5p, miRNA-223-3p, miRNA-21-5p, let 7i-5p, miRNA-29a-3p, and miRNA-210-3p in the LoSc patients. The miRNAs may probably serve as novel biomarkers of LoSc. However, a long-term longitudinal studies are crucial to confirm their prognostic role in the disease.

## Methods

### The study groups

The study included 38 Caucasian patients with LoSc (31 (81.58%) females and 7 (18.42%) males) aged 46.5 ± 20.5 years on average, hospitalized at the Department of Dermatology, Venereology and Pediatric Dermatology of the Medical University of Lublin between 2017 and 2018, as well as 25 healthy volunteers (HVs): 23 (92%) females and 2 (8%) males, aged 54.8 ± 19.7 years on average.

Medical history, including the duration of LoSc, possible triggering factors, and family history was taken. The presence of any extracutaneous manifestations (arthritis, joint pain, contractures, headache, symptoms of eye and nervous system involvement) as well as concurrent autoimmune diseases (Hashimoto’s thyroiditis, vitiligo, lichen planus) were noted.

The exclusion criteria included coexistence of other autoimmune disorders manifested by fibrosis of different organs, i.e. systemic sclerosis, keloids, hypertrophic scars, cardiomyopathy, pulmonary fibrosis, liver fibrosis, and renal fibrosis.

The study protocol was approved by the Bioethics Committee of the Medical University of Lublin and the study was performed in accordance with the relevant guidelines and regulations. All subjects provided written informed consent prior to study enrolment.

### Assessment of localized scleroderma

In the studied LoSc patients, four clinical types of the disease, i.e. generalized, linear, circumscribed, and mixed, were identified. None of the patients had a deep type of LoSc. (classification of LoSc according to the guidelines of European Dermatology Forum, 2017). At the time of inclusion, data on the severity and symptoms of the disease and laboratory parameters, according to the Localized Scleroderma Assessment Tool (LoSCAT), were collected^4^.

The presence of clinical features, i.e. sclerotic areas, erythematous patches without sclerosis, patches with lilac ring, hyperpigmented patches, atrophic patches, and pruritus, was assessed.

The active disease was defined as the appearance of a new erythematous lesion and/or sclerotic plaque or enlargement of the existing inflammatory lesions (erythema and/or lilac ring) during the preceding month. The non-active disease was defined as the presence of hyperpigmentations or discolourations and/or atrophic patches of the skin over at least 6 months. The site of skin lesions, as well as their distribution, were assessed.

Two indexes, i.e. Localized Scleroderma Skin Severity Index (LoSSI) for the assessment of disease activity/severity and Localized Scleroderma Skin Damage Index (LoSDI) for the assessment of tissue damage, were calculated. An extent of the skin lesions was assessed with the use of BSA. The clinical characteristics of the LoSc patients are presented in Table 3.

**Table 3:** Clinical characteristics of the localized scleroderma patients.

Variable	All LoSc patients (N = 38)	Female LoSc patients (N = 31)
Positive family history of LoSc, n (%)	1 (2.63), 0.5–27	0.0
LoSc duration (years), min–max, M ± SD	0.5–27, 5.6 ± 5.2	0.5–27, 5.8 ± 5.4
**Clinical type of LoSc, n (%)**		
Generalized	14 (36.84)	13 (41.94)
Linear	7 (18.42)	4 (12.90)
Circumscribed	10 (26.32)	7 (22.58)
Mixed	7 (16.22)	7 (22.58)
BSA (%), min–max, M ± SD	0.5–50, 8.3 ± 9.2	0.5–50, 9.1 ± 9.9
mLoSSI, min–max, M ± SD	0–50, 10.2 ± 12.1	0–39, 9.4 ± 11.0
LoSDI, min–max, M ± SD	1–45, 10.2 ± 9.3	1–45, 10.6 ± 10.0
Active LoSc, n (%)	27 (71.05)	21 (67.74)
Sclerotic areas, n (%)	28 (73.68)	23 (74.19)
Pruritus, n (%)	6 (15.79)	5 (16.13)
Erythematous patches without sclerosis, n (%)	12 (31.58)	9 (29.03)
Lilac ring, n (%)	14 (36.84)	12 (38.71)
Hyperpigmented patches, n (%)	25 (65.79)	22 (70.97)
Atrophic patches, n (%)	36 (94.74)	29 (93.55)
Extracutaneous manifestations, n (%)	29 (76.32)	25 (80.65)
Concurrent autoimmune diseases, n (%)	8 (21.05)	7 (22.58)
**Possible trigger factor, n (%)**		
Yes	10 (26.32)	7 (18.42)
Only stress	7 (18.42)	7 (18.42)
Only trauma	2 (5.26)	0
both (stress and trauma)	1 (2.63)	0
**Localization of skin lesions, n (%)**		
Face	9 (23.68)	7 (22.58)
Neck	5 (13.16)	4 (12.90)
Trunk	27 (71.05)	24 (77.42)
upper extremities	18 (47.37)	16 (51.61)
lower extremities	26 (68.42)	21 (67.74)
**Distribution of lesions, n (%)**		
Unilateral	14 (36.84)	11 (35.48)
Bilateral	24 (63.16)	20 (64.52)
Linear	15 (39.47)	13 (41.94)
Blaschkoid	2 (5.26)	2 (6.45)
**Current treatment, n (%)**		
Without therapy or topical treatment only	7 (18.42)	5 (16.13))
UV-therapy only	15 (39.47)	12 (38.71)
Immunosuppressive treatment only	6 (15.79)	4 (12.90)
Both UV-therapy and immunosuppressive treatment	10 (26.32)	10 (32.26)
ESR abnormal, n (%)	9 (23.68)	7 (22.58)
CRP abnormal, n (%)	5 (13.16)	4 (12.90)
ANA positive, n (%)	22 (57.89)	18 (58.06)
Rheumatoid factor positive, n (%)	5 (13.16)	3 (9.68)

*M* mean, *SD* standard deviation, *LoSc* localized scleroderma, *CRP* C-reactive protein, *ESR* erythrocyte sedimentation rate, *ANA* antinuclear antibodies, *RF* rheumatoid factor, *mLoSSI* modified Localized Scleroderma Skin Severity Index, *LoSDI* Localized Scleroderma Skin Damage Index, *BSA* body surface area, *UV* ultraviolet, Deep clinical type of LoSc was not observed in any patient.

### Laboratory parameters in the studied localized scleroderma patients

In all the studied LoSc patients, routine laboratory tests, i.e., C-reactive protein (CRP), erythrocyte sedimentation rate (ESR), antinuclear antibodies (ANA) and rheumatoid factor (RF), were performed.

### miRNA isolation

Whole blood (5–10 mL) was collected in BD Monovette plastic tubes (SARSTEDT, Germany). The samples were stored on ice and processed within 1 h of the draw. Serum was isolated by centrifugation at 3000×*g* for 10 min at 4 °C and stored at − 80 °C, in the absence of freeze–thaw cycles, until analysis. Before RNA extraction, we checked the serum samples to test for hemolysis by measuring the absorbance of free hemoglobin at 414 nm, and samples with OD_414_ greater than 0.2 were excluded^77^, due to the potential of cellular miRNAs to confound the results. RNA was extracted from 200 µL serum using the Syngen miRNA Mini Kit (Syngen, Poland; SY391210) following the manufacturer’s protocol. With this kit it is possible to isolate total RNA and miRNA, thus, we were able to evaluate the concentration of both fractions. Briefly, after mRL buffer (200 µL) was added and mixed, the samples were incubated at room temperature for 10 min, the addition of 450 µL phenol and 200 µL chloroform was useful to achieve aqueous and organic phase separation. In the next step, the aqueous phase was applied to an RNeasy spin column. The purified RNA was eluted in 50 µL mRE buffer and stored at − 80 °C.

### Analysis of miRNA quality and integrity

miRNAs’ concentration, A260/230, and A260/280 ratios were evaluated by NanoDrop UV/Vis spectrophotometer (2000, ThermoFisher SCIENTIFIC, Waltham, MA, USA). Later, two different chips: Agilent RNA 6000 Nano Kit for total RNA and Agilent Small RNA kit for low molecular weight RNA were used to check the quality and the integrity of total and small RNA. It was measured by capillary electrophoresis with 2100 Agilent Bioanalyzer (Agilent Technologies, Santa Clara, CA, USA). Electropherograms were visualized with the Agilent 2100 Expert software.

### Pathway-focused miScript miRNA PCR arrays

To find the biologically relevant pathway-focused, disease-focused or whole miRNome panels in our patients’s samples, the SYBR Green-based real-time PCR profiling of miRNAs with the miScript PCR System (miScript miRNA PCR Array Human miFinder Pathway- or disease-focused panels in 96-well, QIAGEN, Germany; 331221-MIHS-001ZD-2) was used following the manufacturer’s protocol. Briefly, the isolated total RNA was reverse transcribed to cDNA using a Superscript II First Strand Synthesis System (QIAGEN). Each reverse transcriptase (RT) reaction contained 125–250 ng of the RNA sample, 2 µL of 10 × miScript Nucleic Mix, 2 µL of 10 × miScript Reverse Transcriptase Mix, and 4 µL 5 × miScript HiSpec Buffer. The total was 20 µL. The analyses were performed in thermal cycler C1000 (BIO-RaD, Hercules, CA, USA) for 60 min at 37 °C, followed by a heat-inactivation step for 5 min at 95 °C and held at 4 °C.

In the next step, the serum levels of a panel of 84 miRNAs were assessed according to the manufacturer’s protocol, along with three control sets present in this panel; the first contained six miRNAs (snoRNA/snRNA) whose average of readings enables normalization of the array data using the relative quantification method, the second was used for assessing the performance of reverse transcription reaction, and the third was used for assuring PCR performance. qPCR analysis was performed in 96-well plates using the CFX96 real-time PCR detection system (BIO-RAD, Hercules, CA, USA) in conditions as follows: initial activation step at 95 °C for 15 min, 50 cycles of denaturation at 94 °C for 15 s followed by an annealing step at 55 °C for 30 s, then extension step at 70 °C for 30 s. The relative miRNAs’ serum levels were calculated using the 2^−ΔΔCt^ method. The data were presented as the fold change in gene expression normalized to an endogenous reference gene (RNU48) and relative to the control (the HVs)^78^.

### Target gene analysis

In our study, we attempted to identify the miRNAs’ levels in LoSc patients compared to healthy volunteers (HVs) using Pathway-focused miScript miRNA PCR Arrays analysis (Table 1). Having done the bioinformatics analysis, we have selected 6 miRNAs. Therefore, we validated the chosen miRNAs in the group of LoSc patients using RT-qPCR assay.

We used target gene prediction of the selected miRNAs and tried to check their potential role in LoSc pathogenesis. Relevant target genes were identified by using databases Target Scan v7.1 (https://www.targetscan.org)^79^, which predicts miRNAs biological targets, miRDB v4.0 (https://mirdb.org/miRDB/)^80^, and miRSearch v3.0 (https://www.exiqon.com/miRSearch). Moreover, we performed pathway analysis by DNA Intelligent Analysis (DIANA)-miRPath v5.0 software, based on the data from Ensembl v69 and miRBase v18 (https://diana.imis.athena-innovation.gr)^81^, which uses miRNA targets based on DIANA-microT-CDS, and predicts miRNA-gene interaction.

### Quantitative RT-PCR (RT-qPCR) of selected miRNAs

To confirm the results obtained by miRNA PCR array, we performed quantitative real-time PCR. miRNAs’ serum levels were measured in all 63 samples (38 patients and 25 healthy controls) using the TaqMan MicroRNA Reverse Transcription Kit (Applied Biosystems, Foster City, CA, USA). In the first step, total RNA (25 ng) was used for reverse transcription with TaqMan Universal PCR Master Mix (Applied Biosystems) and TaqMan probes (Applied Biosystems) in 96-well plates using the CFX96 real-time PCR detection system (BIO-RAD, Hercules, CA, USA) following the manufacturer’s protocol. Specific miRNAs primers were used for miRNA-223-3p, miRNA-210-3p, let 7i-5p, miRNA-21-5p, miRNA-29a-3p (Life Technologies, Carlsbad, CA, USA) and 181b-5p (Applied Biological Materials Inc. (abm good), Canada). Mature miRNA sequences and their Assay ID were used as follows:

- miR-223-3p—UGUCAGUUUGUCAAAUACCCCA, 002295,
- miR-210-3p—CUGUGCGUGUGACAGCGGCUGA, 000512,
- let-7i-5p—UGAGGUAGUAGUUUGUGCUGUU, 002221,
- miR-21-5p—UAGCUUAUCAGACUGAUGUUGA, 000397,
- miR-29a-3p—UAGCACCAUUUGAAAUCGGUUA, 000412,
- miR-181b-5p—AACAUUCAUUGCUGUCGGUGGGU, MPH02242.

All the PCR reactions were run in 3 replicates. The Ct values were normalized to RNU48 for miRNAs. Gene expression was analyzed using the difference in cycle threshold (ΔCt) method using the manufacturer’s software (BIO-RAD). The relative miRNAs’ serum levels were calculated using the 2^−ΔΔCt^ method and presented as the fold change of the control (HVs).

### Statistical analysis

The data were statistically analyzed using STATISTICA 13 software. Minimum and maximum values, mean (M) and standard deviation (SD) or median and interquartile range (IQR) were estimated for continuous variables, as well as absolute numbers (n) and percentages (%) of the occurrence of items for categorical variables. All the analyses were conducted firstly for all the patients (both female and male), and secondly only for the female patients.

We used statistical tests as follows: Mann–Whitney’s U test (to compare age as well as the selected miRNAs’ levels in the serum between LoSc patients and HVs), χ^2^ test (to compare gender between the LoSc patients and the HVs), Mann–Whitney’s U test (to compare the selected miRNAs’ serum levels between two groups of LoSc dichotomous characteristics), Kruskal–Wallis’ H test (to compare the selected miRNAs’ levels in the serum between three or more groups of LoSc polytomous characteristics), Spearman’s correlation coefficient r (to correlate the selected miRNAs’ serum levels with LoSc continuous characteristics).

Since miRNA levels for both groups of patients were assessed relative to the control, in case of significant differences in miRNA levels between the two groups of LoSc dichotomous characteristics, we calculated the relative fold change between two groups. We divided the relative fold change for the first group by the relative fold change for the second group where the former was the group with a higher relative fold change relative to the control.

The significance level was assumed at 0.05.
